# Influence of Delamination Size and Depth on the Compression Fatigue Behaviour of a Stiffened Aerospace Composite Panel

**DOI:** 10.3390/polym15234559

**Published:** 2023-11-28

**Authors:** Angela Russo, Rossana Castaldo, Concetta Palumbo, Aniello Riccio

**Affiliations:** Department of Engineering, University of Campania Luigi Vanvitelli, Via Roma 29, 81030 Aversa, Italy; angela.russo@unicampania.it (A.R.); rossana.castaldo@studenti.unicampania.it (R.C.); concetta.palumbo@unicampania.it (C.P.)

**Keywords:** stiffened panel, circular delamination, structural design, finite element analysis

## Abstract

Delamination in reinforced panels is one of the primary challenges facing the safety and reliability of aerospace structures. This article presents a sensitivity analysis of the fatigue behaviour during the compression of a composite aeronautical stiffened panel experiencing delamination. The main objective is to assess the impact of delamination size and depth on the lifecycle and structural integrity of the panel. Different dimensions and positions of delamination are considered to cover a comprehensive range of damage scenarios. The key feature of this sensitivity analysis is the adoption of a numerical procedure that is mesh- and load-step-independent, ensuring reliable results and providing valuable insight into the criticality of delamination and its impact on the fatigue behaviour during the compression of reinforced aeronautical panels. Sensitivity analyses are essential for enhancing the design process of aerospace structures, thereby contributing to the increased safety and reliability of structural components. In this regard, the use of robust and effective numerical procedures is of crucial significance. This may be seen as the real added value of this paper.

## 1. Introduction

Fracture has always been a significant issue for the aerospace industry during the fabrication of aerospace structures, and, over the years, it has become more severe due to the extensive use of innovative materials, such as composite materials [1,2], which are very prone to interlaminar fractures [3]. To take full advantage of the benefits offered by these materials, they are used to fabricate stiffened panels that are used in the aircraft fuselages, wings, and horizontal/vertical tail planes. However, the latter, when in service, are exposed to impact phenomena, which can cause extensive damage, sometimes not visible on first visual inspection, leading to catastrophic events [4,5,6]. Moreover, manufacturing flaws and maintenance processes may cause the onset of cracks that can easily propagate under service loads [7,8]. The phenomenon of fatigue amplifies these issues, significantly reducing the strength properties of stiffened composite aircraft components through material degradation [9,10]. 

Many studies have been carried out in the literature to consider the delamination phenomenon due to a variety of causes. The study in [11] introduced a mesh-independent computational method, utilising the extended finite element methodology, to predict transverse matrix crack and delamination evolution. The approach demonstrated good agreement with conventional numerical methods and experiments. In [12], a review on how delamination contributes to the failure of fibre-reinforced composites is presented. The study in [13] explored hydrothermal aging in a carbon-fibre-reinforced laminate and its epoxy matrix in bulk conditions. Minimal changes in bulk resin fracture toughness but variable trends in composite interlaminar fracture toughness were observed. In [14], composite laminates impacted at low velocities were studied. Drop weight tests and ultrasonic C-scan were employed to assess delamination evolution. Numerical modelling, including the cohesive contact method, has been used to simulate delamination. The FEM results have been proven to be aligned with experiments. Liu et al. [15] conducted a 2D and 3D parametric finite element analysis of composite flat laminates with two types of through-the-width delamination. The effect of multiple delaminations on postbuckling properties has been studied by using the virtual crack closure technique. The study in [16] employed Abaqus, enhancing fatigue analysis by integrating R-curve effects into the Paris law through an empirical method. Implemented via a user-defined subroutine, the novel method accurately predicts fatigue life within 5% of the test results. The work in [17] explored disbonding in adhesively bonded panels through Lamb waves during fatigue tests, emphasising frequency, mode, sensor placement, and parameter selection. Piezoelectric transducers act as actuators and sensors, correlating crack propagation rate with A0 mode velocity. Šedková et al. [18] investigated debonding and delamination assessment by means of Lamb waves in adhesively bonded composite joints.

The study of damage propagation in aircraft structures must begin at the preliminary design stage. Indeed, the use of new design concepts and innovative materials, whose damage behaviour is not well understood, often leads to the over-dimensioning of aircraft structures. Furthermore, in a damage-tolerant design philosophy, the loads and stresses that the aircraft is expected to withstand in operation must be considered in detail, and the effects of the damages on the structural integrity of the component must be assessed. The work in [19] delved into the numerical methods employed to forecast impact damage in advanced composite structures, emphasising their significance for industry approval in designing and certifying composite aircraft. The article in [20] addresses the low-velocity impact (LVI) challenge in the composites industry, highlighting how factors are crucial for improving the impact resistance and damage tolerance of fibre-reinforced composites (FRCs). The review identifies gaps in the literature and suggests future research directions, while also discussing various damage modelling strategies to predict the impact resistance and damage tolerance of FRCs. Jones [21] addressed challenges related to aging aircraft and infrastructure, emphasising the need for tools to predict crack growth from small material discontinuities. They discuss the differences in the analysis tools used for design and sustainment, modelling crack growth, determining short crack data, predicting growth using existing equations, and accounting for variations in crack growth histories. In [22], the damage tolerance of a stitched carbon/epoxy laminate was studied. Investigating the impact of stitching on a carbon fibre epoxy laminate revealed significantly improved damage tolerance compared with that of brittle epoxy.

Appropriate experimental testing campaigns should be conducted to identify structural weaknesses. However, the production of aircraft structures, even on a small scale, to identify the best structural configurations can be very expensive as well as time-consuming. Aircraft designers and engineers use a variety of tools and techniques to analyse and predict the behaviour of materials and structures under operating loads, including finite element analyses [23,24,25,26,27,28]. 

Sensitivity analyses are essential in structural design and projects to identify the critical geometrical parameters that have the most significant impact on the structural design or project outcome [15,29,30,31]. By adapting these variables (within a reasonable range), engineers can understand which factors play a crucial role in the performance and damage behaviour of a structure. Moreover, sensitivity analysis allows engineers to evaluate the robustness of the design against variations in input parameters. In general, sensitivity analyses provide valuable insights into the behaviour and performance of structural configurations. This helps designers make proper decisions, optimise designs, manage risks, and ensure the structural integrity and efficiency of the final product.

The main added value of this article is the use of the FT-SMXB methodology, due to its inherent characteristics, to study, specifically, the influence of the size and depth of typical impact damage on composite panels. The standard virtual crack closure technique (VCCT) would probably influence the results due to its dependence on the mesh and time step of the finite element analysis. On the contrary, one of the advantages of the cohesive zone model (CZM) methodology is its lower sensitivity to mesh size. However, defining the correct cohesive stiffness is still a challenge [32], and a rigorous calibration of the cohesive parameters is needed to avoid mesh sensitivity [33]. For the finite element method, the dependence of the results on the size of the elements selected for the numerical model discretisation and the timestep used for the load application during the analyses play a key role in the obtained results. The FT-SMXB numerical approach has been proven to overcome these FEM limitations. This procedure has been validated both at the coupon level, such as in the double cantilever beam test, end-notched flexure test and single leg bending shear test, and for more complex structures, such as stiffened panels characterised by skin-stringer debonding [34,35]. The use of this numerical procedure is the real added value of this work, which includes a sensitivity analysis of a typical aeronautical reinforced panel subjected to typical impact damage of varying size and depth in the skin panel bay. This manuscript can be considered an evolution of previous work [36], where the static failure of an aeronautical stiffened composite panel under compressive loading conditions was studied. The results showed a strong dependence of the panel compressive behaviour on the geometric parameters, including the depth and size of the circular delamination. Here, the fatigue behaviour of the panel was assessed by varying the radius and the position in the thickness of a circular delamination. The influence of such variables on the delamination evolution and the stiffness degradation was assessed. The delamination between two interfaces was considered. The FT-SMXB procedure does not consider the delamination migration phenomenon.

## 2. Numerical Model Description

A typical aeronautical stiffened panel was studied under compressive fatigue loading conditions. The panel was 300 mm in length and 400 in width. Two T-shape stringers were tied on the skin, which has a 60 mm foot and 30 mm web. The stacking sequences of the different panel components, which are listed in Table 1, were chosen according to typical manufacturing process needs. A 0.165 mm ply of a carbon fibre/epoxy resin material system was used to model the thicknesses of the panel parts. Circular delamination damage in the panel bay was placed, representing a typical impact damage. It is known that the shape of delamination after impact in composite structures can change depending on several factors, including the nature and extent of the impact, the material properties, and the geometry of the structure. While circular delamination is commonly used in theoretical and analytical models for simplicity, real delamination resulting from impacts can have different shapes. However, in some cases, the delamination may have a circular or nearly circular shape, especially if the impact is relatively symmetrical and occurs at a specific point.

**Figure 1:**
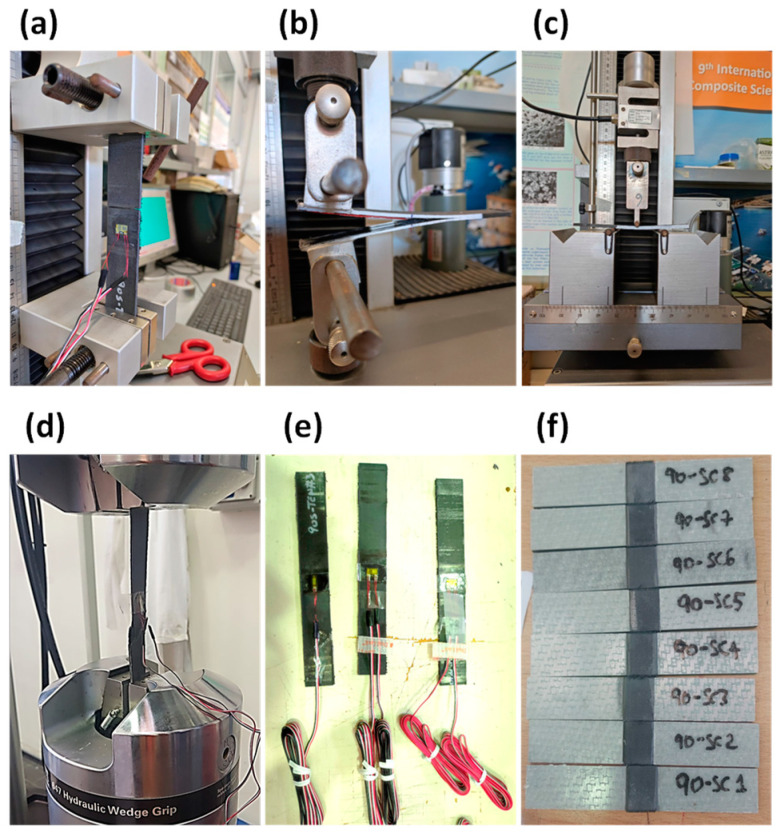
Experimental characterisation of the material: (**a**) matrix tensile test; (**b**) double cantilever beam test; (**c**) end-notched flexure test; (**d**) fibre tensile test; (**e**) matrix tensile specimens; (**f**) fibre compressive specimens.

The elastic and interlaminar properties of the material were evaluated through an experimental characterisation, based on the ASTM standards in [37,38,39,40,41,42], and are reported in Table 2. Static tensile, compressive, and shear tests were performed on samples manufactured according to the standards. The tests summary is shown in Figure 1.

**Figure 2:**
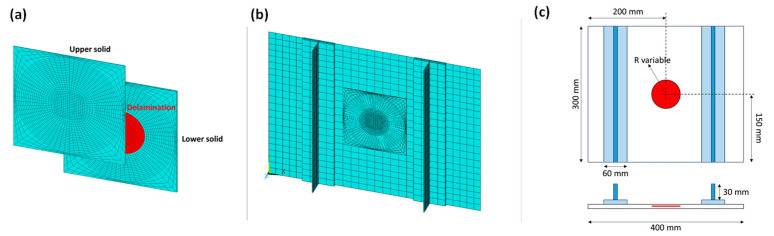
(**a**) Fail release approach; (**b**) FEM model of the panel; (**c**) schematic of the panel.

The panel was modelled through the Ansys Parametric Design Language (APDL) to easily modify the value of the variable parameters (radius and depth). The global–local approach was chosen as modelling strategy in order to reduce the computational cost and the analyses’ duration. Indeed, a coarse mesh was considered for the global part, while a more refined discretisation was performed in the local zone, which is the region characterised by the delamination damage and is involved in the propagation. Elements of 12.5 mm were used for the global model, while elements of approximately 2 mm and 5 mm were considered in the local region. According to the fail release approach, typically used in conjunction with the virtual crack closure technique (VCCT), the local zone was built with two overlapping identical solids, as shown in Figure 2a. Identical discretisation was carried out, and the nodes, which are characterised by the same spatial coordinates, were connected with constraints with “birth and death” capabilities, except in the delaminated area, where nodes are released, and only interactions were considered to avoid penetration. The FEM model is displayed in Figure 2b. A schematic of the panel is reported in Figure 2c.

**Figure 3:**
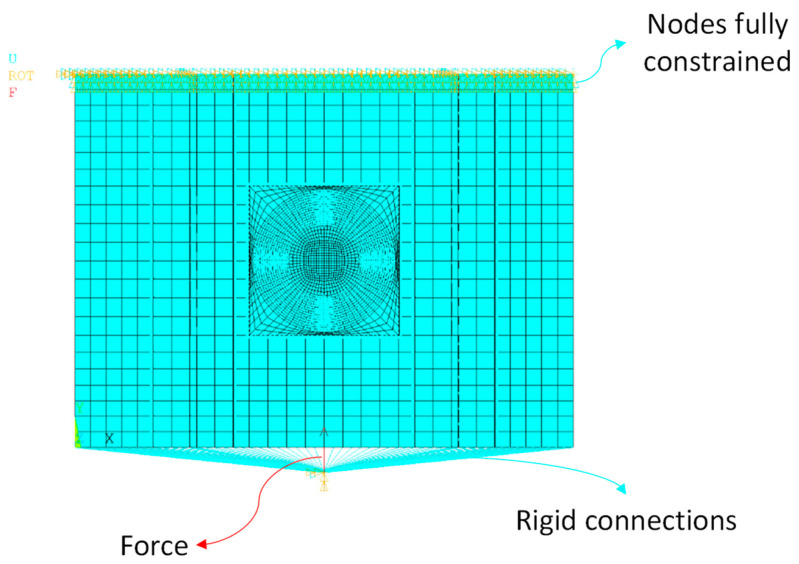
Boundary conditions of the fatigue analyses.

The translational and rotational degrees of freedom on one side of the panel were clamped (interlocking constraint); on the other side, the terminal nodes were connected through rigid connections to a reference point (pilot node) on which the compressive force was applied. The scheme of the boundary conditions is shown in Figure 3.

### 2.1. FT-SMXB Approach

Fracture mechanics relations are the basis of the FT-SMXB numerical method. In particular, the modified virtual crack closure technique (VCCT) equations were implemented to calculate the energy release rate (ERR) values on the nodes of the delamination front. Such values were compared with the critical fracture toughness values to assess the propagation of delamination, as described by Equation (1).

(1) $$\frac{G_{I}}{G_{Ic}}+\frac{G_{II}}{G_{IIc}}+\frac{G_{III}}{G_{IIIc}}=E_{d}\geq 1$$

**Figure 4:**
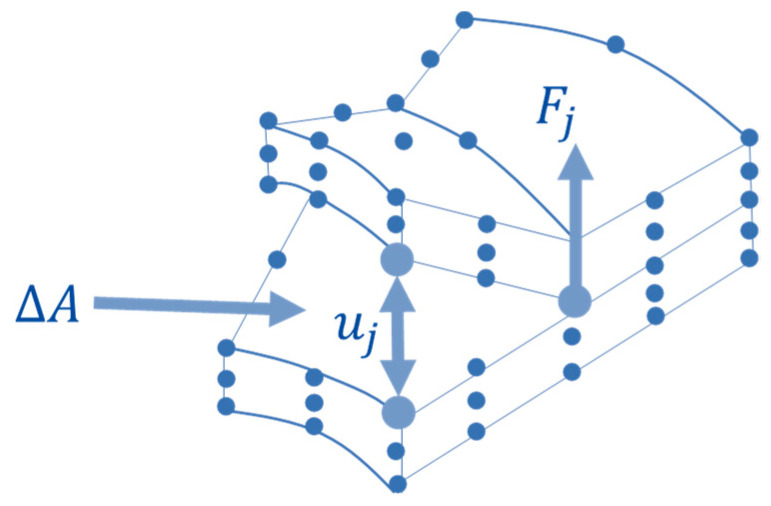
Crack tip.

The ERR $G_{j}\;$with j=I,II,III depends on the increase in delaminated area ∆A, as reported in Equation (2), where $F_{j}$ is the force at the crack tip, and $u_{j}$ is the opening displacement (see Figure 4).

(2) $$G_{j}=\frac{F_{j}u_{j}}{2∆A}\;with\;j=I,II,III$$

According to the VCCT, $G_{j}\;$is the amount of energy needed to close an area ∆*A* of the fracture surface, while $G_{jc}\;$is the critical value that the energy must reach to open the crack. However, considering the FEM approach, the delamination growth is influenced by the chosen element size. Therefore, even if, at a specific load step, the delaminated area is smaller than that of an entire element (for the computed energy values), it is overestimated.

In the FT-SMXB approach, the VCCT limits in terms of mesh and dependence on load step size are avoided through the implementation of an iterative procedure, named SMART TIME, that can iteratively change the load step size until the calculated energy corresponds exactly to the amount needed to propagate an area equal to that of one or more elements. In detail, the equivalence in Equation (3) must be satisfied. The subscript *i* refers to the nodes of the delamination front.

(3) $$\sum_{i=1}^{N}A_{i}=\sum_{i=1}^{N}\left(\sum_{j=1}^{3}\frac{F_{ji}^{\left(t+1\right)}u_{ji}^{\left(t+1\right)}}{2G_{jc}}\right)≅\sum_{i=1}^{N}A_{i}^{Element}$$

Cyclic load can be applied on the structure via a succession of static analyses, which can be considered as fatigue cycles. The load applied on the structure in each static analysis is calculated as a percentage of the maximum load that the structure can support when subjected to static loading conditions. Basically, a first static analysis is performed, and the ERR values on the delamination front nodes are calculated and used as input in the Paris law criterion, defined by Equation (4). *N* represents the number of cycles, while *C* and *n* are the Paris law constants evaluated experimentally.

(4) $$\frac{∆A_{i}}{∆N_{i}}=C\;f{\left(G\right)}^{n}$$

The number of cycles needed to propagate delamination damage is calculated by reversing the Paris law, as described in Equation (5). Finally, the node with the higher criterion value, which is the node where the energy values are closest to the critical values, is selected and released.

(5) $$∆N_{i}=\frac{∆A_{i}^{t}}{C{f\left(G\right)}^{n}}$$

Starting from the last converged solution, subsequent analyses are performed until the number of cycles to failure is reached, or until the user-defined limits are encountered. Damage accumulation is considered. A damage variable is considered in each node of the delamination front to account for damage accumulation, and the number of cycles starts from that reached in the previous analysis.

### 2.2. Preliminary Static Analysis

Three different radii of circular delamination were studied: 20 mm (R20), 30 mm (R30), and 40 mm (R40). Each of them was positioned at three depths along the thickness (see Table 3). This placed the delamination between plies of different orientations. Specifically, under two laminae (2PLY), the delamination was between 0 and 90 degrees; under three laminae (3PLY), the delamination was between 90 and 45 degrees; and under four laminae (4PLY), it was between 45 and −45 degrees.

**Figure 5:**
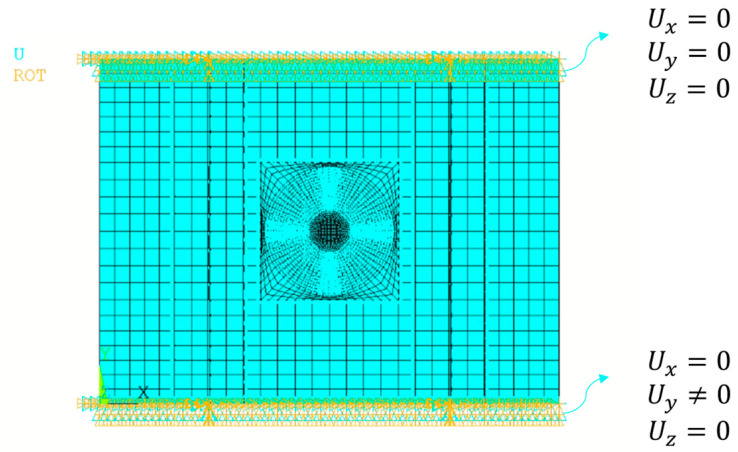
Boundary conditions of the static analyses.

The propagation initiation load and the overall buckling behaviour of the panel under static loading conditions were needed to proceed with fatigue analyses. Hence, preliminary analyses were conducted considering the panel with one edge fixed (all degrees of freedom constrained) and the other subjected to compressive displacement (free degrees of freedom in the compression direction). Figure 5 shows the boundary conditions. 

**Figure 6:**
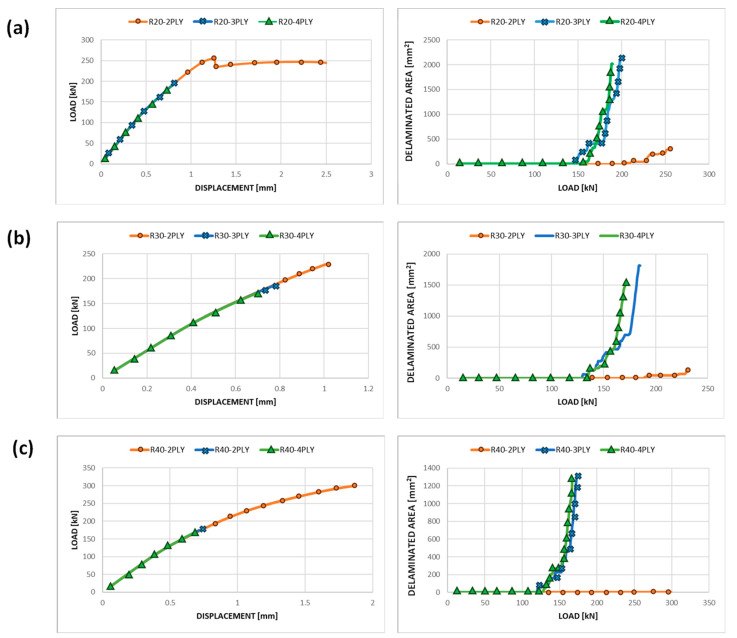
Load vs. applied displacement and delaminated area vs. load curves—static analyses: (**a**) R20 panel; (**b**) R30 panel; (**c**) R40 panel.

Figure 5 shows the load as a function of applied displacement curves and the trend of delaminated area with the load for each considered configuration. The charts in Figure 6 indicate that increasing the depth of delamination, with the same radius, reduces the load supported by the structure, and the propagation of delamination becomes unstable and rapid, leading to sudden delamination evolution. The ultimate displacement applied to the structure, visible in Figure 6, does not represent its point of failure but only the displacement applied in the last step of the analysis. The end of the simulation is due to the achievement of the maximum extent of delamination, which is limited to the circular region around the initial defect. 

**Figure 7:**
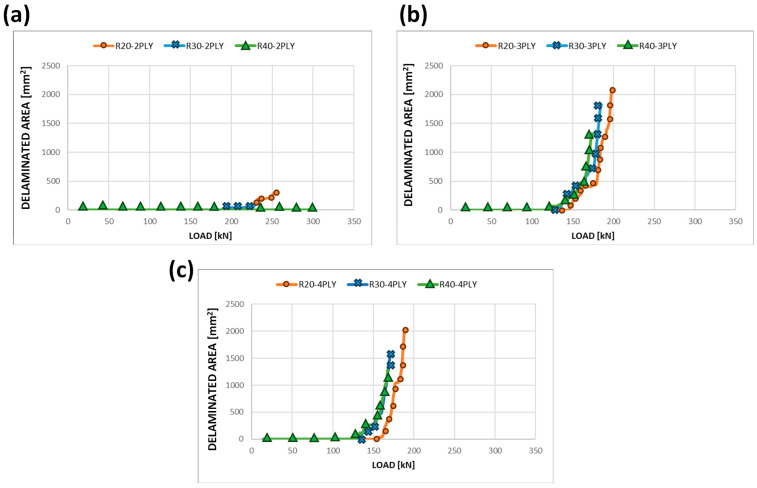
Delaminated area vs. load curves at fixed delamination depth—static analyses: (**a**) 2-ply depth of delamination; (**b**) 3-ply depth of delamination; (**c**) 4-ply depth of delamination.

Fixing the position along the thickness of the delamination damage, as the radius increases, there are no significant variations in the delaminated area trend with increasing load; as demonstrated in Figure 7, only a slight increase in the speed of propagation can be noticed for larger radii.

**Figure 8:**
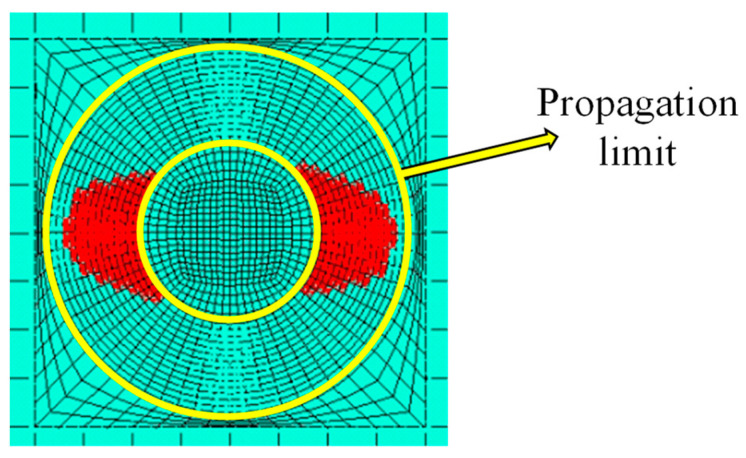
Delamination propagation limit.

It is worth specifying that the analyses automatically terminate when the delamination (and thus, the nodes released using the fail release approach) reach the user-defined limit. In this specific case, the limit was set at the outmost nodes of the circular propagation crown considered, as shown in Figure 8.

Based on the obtained results, considering the load at which delamination starts propagating, fatigue analyses were conducted, according to Table 4.

## 3. Results and Discussion

The summary of the performed fatigue analysis is reported in Table 4. The configuration R20/30/40 2P was not considered for fatigue assessment because the results in Figure 7 show that a slight propagation of the damage is experienced by the panel, which undergoes global buckling by preventing the growth in the delamination.

**Figure 9:**
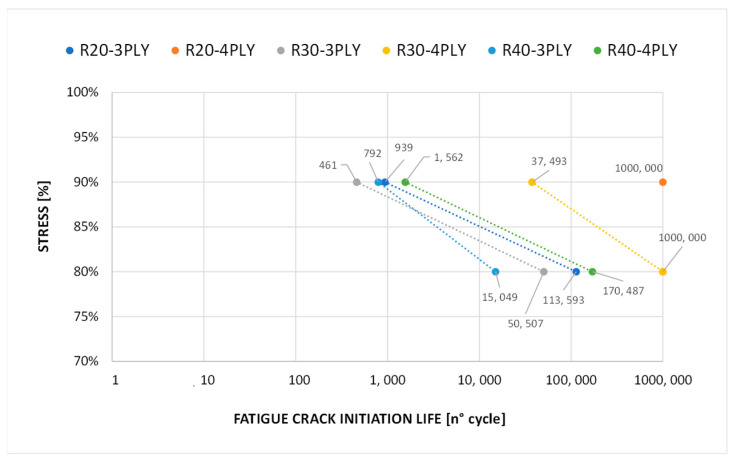
Fatigue crack initiation life curves.

The fatigue damage initiation life refers to the duration or number of loading cycles that a material or structure can undergo before the initiation of fatigue damage. Evaluating fatigue damage initiation life involves analytical methods, such as cycle counting methods and the damage accumulation model, which are taken into account in FT-SMXB. The delamination initiation cycle was determined through the Paris law relation, as defined in Equation (5). Figure 9 shows a graph of the fatigue crack initiation life values for the various configurations analysed at 90% and 80% of the static delamination initiation load.

**Figure 10:**
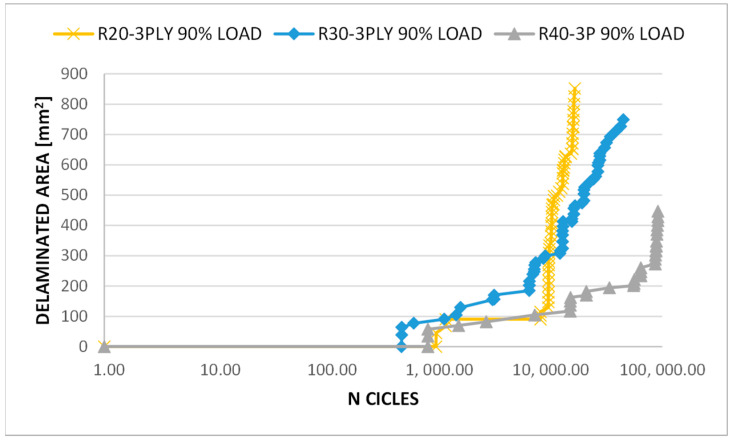
Delaminated area vs. number of cycles (log-scale)—fatigue analyses.

Figure 10 displays the delamination evolution over as a function of the number of cycles for three different radii of circular delamination under three plies. According to the curves’ trend, the larger delamination size (R40-3PLY) resulted in better fatigue behaviour of the composite panel. Indeed, a smoother growth of the delaminated area can be seen, achieving a higher number of cycles, although with a lower value of total delaminated area. In fact, in some specific cases, a larger delamination in a composite panel may create a more gradual stress transition in the surrounding area, which means that there are lower stress gradients in the material surrounding the delamination, thus reducing stress concentrations and the probability of further cracking and propagation.

**Figure 11:**
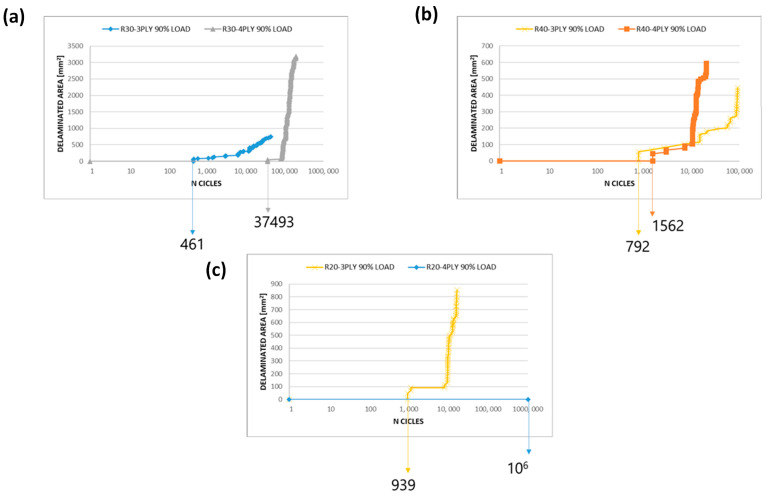
Delaminated area vs. number of cycles (log-scale)—fatigue analyses: (**a**) R30 configuration; (**b**) R40 configuration; (**c**) R20 configuration.

Increasing the depth of delamination for the same radius can have complex effects on the fatigue behaviour of a composite panel. According to Figure 11, increasing the depth of the delamination can increase the onset cycle, which is the number of cycles required for the crack to initiate. The relationship between delamination depth and fatigue behaviour is typically not linear. Primarily, deeper delamination could delay crack initiation due to the higher load required to propagate the crack deeper into the material [43,44]. However, as the delamination extends, it becomes a more significant structural defect, leading to higher stress concentrations and potentially accelerated crack growth.

**Figure 12:**
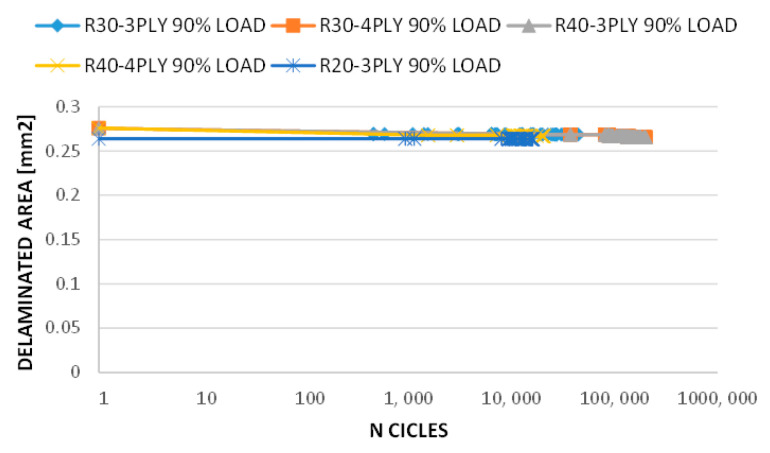
Stiffness vs. number of cycles (log-scale)—fatigue analyses.

According to Figure 12, the stiffness (defined as load/displacement and measured on the nodes of load application) of all the analysed configurations decreased by less than 5% because, in the considered cases, the delamination extension is limited and hence does not affect the overall stiffness of the structure. The investigated structures maintain their overall integrity, with relatively small changes in stiffness within an acceptable range. Even if the stiffness remains almost constant, different levels of damage size and depth can influence the stress intensity in the damaged region. This can affect the number of loading cycles required for crack initiation and the rate of crack growth during propagation.

**Figure 13:**
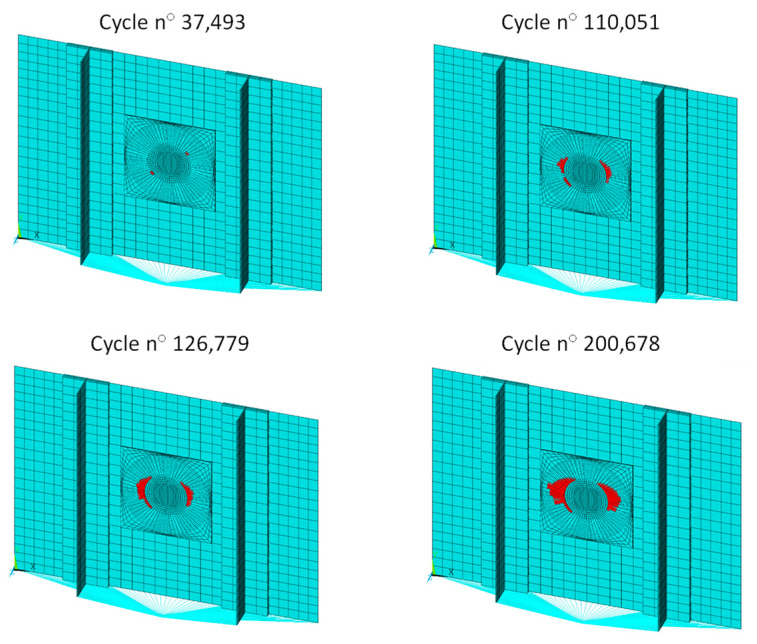
Evolution of the damage for different numbers of cycles.

The evolution of delamination for different numbers of cycles is shown in Figure 13 for one of the analysed configurations (R30 4PLY), as an example. The red region indicates the elements that correspond to the nodes released by the VCCT, which reached complete failure according to the Paris law calculations. Therefore, the red portion represents the growth achieved in the delaminated area.

## 4. Final Remarks

In this work, the mesh- and load-step-independent FT-SMXB numerical methodology was employed to assess the compressive fatigue behaviour of a composite material, an aeronautical stiffened panel, affected by typical impact damage in the bay region. Two percentages of the static damage initiation load were considered (80% and 90%). The results revealed that for greater radii of the circular delamination, at a fixed damage depth, delaminated areas experience smooth growth, achieving a higher number of fatigue cycles with a lesser damage extent. Furthermore, increasing the depth of the damage, at a fixed radius, increases the delamination onset cycle but leads to faster and unstable delamination evolution. The overall stiffness of the panel remains almost constant with the fatigue cycles for all the considered radii and depths of delamination. 

It is important to note that the specific behaviour of composites and the effect of delamination depend on various factors, including the type of composite material, its layup configuration, the loading conditions, and the size and shape of the delamination. However, the use of effective and robust computational methodologies can provide help in designing damage-tolerant composite structures for various applications and loads, thus reducing the waste of money and time derived from manufacturing and experimental tests. The primary contribution of this article lies in the application of the FT-SMXB methodology, which, owing to its intrinsic characteristics, proves exceptionally valuable in examining the impact of size and depth variations in a typical damage scenario on composite panels. Unlike the standard VCCT and CZM methodologies, which are likely to impact the results due to their reliance on the mesh and time step parameters in finite element analysis, the FT-SMXB methodology stands out for its independence from these factors.

## Figures and Tables

**Table 1:** Stacking sequences.

Component	Number of Plies	Layup	Thickness
Skin panel	16	[45,90,0,−45]_2s_	2.64 mm
Foot stringer	24	[(0,90,90,0)_s_(45,90,0,−45)_2s_]	3.96 mm
Web stringer	8	[0,90,90,0]_s_	1.32 mm

**Table 2:** Material properties.

Property	Value	Description
$E_{11}$	122,000 MPa	Young’s modulus in the fibre’s direction
$E_{22}=E_{33}$	6265 MPa	Young’s modulus in the transverse direction
$G_{12}=G_{13}$	4649 MPa	Shear modulus in the 1–2 and 1–3 planes
$G_{23}$	4649 MPa	Shear modulus in the 2–3 plane
$\nu_{12}=\nu_{13}$	0.3008	Poisson’s ratio in the 1–2 and 1–3 planes
$\nu_{23}$	0.02	Poisson’s ratio in the 2–3 plane
G_Ic_	180 J/m^2^	Mode I critical energy release rate
G_Iic_ = G_IIIc_	1900 J/m^2^	Mode II and Mode III critical strain energy release rate
c1	0.7188	Mode I Paris constant
n1	8	Mode I Paris exponent
c2	6.5938	Mode II Paris constant
n2	6	Mode II Paris exponent

**Table 3:** Panel configurations.

Id Configuration	Delamination Radius	Delamination Depth
R20-2PLY	20 mm	0.33 mm
R20-3PLY	20 mm	0.495 mm
R20-4PLY	20 mm	0.66 mm
R30-2PLY	30 mm	0.33 mm
R30-3PLY	30 mm	0.495 mm
R30-4PLY	30 mm	0.66 mm
R40-2PLY	40 mm	0.33 mm
R40-3PLY	40 mm	0.495 mm
R40-4PLY	40 mm	0.66 mm

**Table 4:** Fatigue analyses.

Id Configuration	Static Delamination Onset Load	Fatigue Loads	Onset Cycle	Failure Cycle
R20-3PLY	147 kN	80% 90%	113,593 939	>1 × 10^6^ 16,459
R20-4PLY	158 kN	80% 90%	>1 × 10^6^ >1 × 10^6^	-
R30-3PLY	130 kN	80% 90%	50,507 461	672,364 44,866
R30-4PLY	135 kN	80% 90%	>1 × 10^6^ 37,493	- 200,678
R40-3PLY	121 kN	80% 90%	15,049 792	>1 × 10^6^ 91,584
R40-4PLY	130 kN	80% 90%	170,487 1562	>1 × 10^6^ 20,665

## Data Availability

Data is contained within the article.
